# Water use strategies of *Nitraria tangutorum *in the lake-basin region of the Badain Jaran Desert

**DOI:** 10.3389/fpls.2023.1240656

**Published:** 2023-08-15

**Authors:** Jie Qin, Jianhua Si, Bing Jia, Chunyan Zhao, Dongmeng Zhou, Xiaohui He, Chunlin Wang, Xinglin Zhu

**Affiliations:** ^1^ Key Laboratory of Eco-Hydrology of Inland River Basin, Northwest Institute of Eco-Environment and Resources, Chinese Academy of Sciences, Lanzhou, China; ^2^ University of Chinese Academy of Sciences, Beijing, China

**Keywords:** water use, water response mechanism, desert lake-basin region, stable isotope, plant water use pattern

## Abstract

Information regarding plant water-use strategies is essential for understanding the hydrological processes and plant survival adaptation mechanisms in desert lake basin regions. To examine the water use strategies of plants in desert lake basin areas, water uptake patterns, water use efficiency, and water potential of *Nitraria tangutorum* were investigated at different distances from the lake duringhe growing seasons in the lake basin regions of the Badain Jaran Desert. The results indicate that *N. tangutorum* primarily absorbed groundwater in May (63.8%) and August (53.5%), relied on deep soil water in June (75.1%), and uniformly absorbed soil water from different layers in July. These observations could be explained by periodic fluctuations in the groundwater level and the consequent decrease in soil water availability, as well as plant root adjustments. As soil water availability decreases, *N. tangutorum* adapts to water variation by increasing its water use efficiency (WUE) and reducing its leaf water potential (*Ψ*). With intensified water stress, *N. tangutorum* gradually shifted from adventurous anisohydric regulation to conservative isohydric regulation. Thus, *N. tangutorum* responds to diverse degrees of environmental changes by altering its water-use strategy. A better understanding of the adaptive water use strategies developed by desert plants under varying water availability conditions provides insight into the diversity of species’ reactions to long-term drought and quantifies the hydrological cycle of desert ecosystems against the background of worldwide climate warming.

## 1 Introduction

Over the past few decades, climate change has become an important worldwide challenge (Huo et al., 2022). Unprecedented increases in mean temperatures and the frequency of severe droughts and heat events (Hartmann et al., 2021) have contributed to the widespread wilting and death of plant populations (Guo et al., 2020; Hartmann et al., 2021; Huo et al., 2022). These phenomena have important implications for ecosystem processes and patterns. Moreover, numerous studies have determined that hydrological space-time variations due to global temperature change have substantial effects on plant-water relationships (Chen et al., 2021; Ding et al., 2021; Zunzunegui et al., 2022). The mechanisms and extent of plant responses to hydrological variations are determined by the water utilization characteristics and water stress adaptations of plants (Grossiord et al., 2017; Wu et al., 2019). Knowledge of plant water-use strategies is important to enhance our understanding of plant responses to hydrological conditions in water-scarce habitats. Conversely, plants, as the central link in the water cycle, are essential for controlling hydrological processes. Accordingly, an accurate understanding of plant water-use strategies under water stress is gaining importance for quantifying the ecosystem hydrological cycle under changing future climatic conditions (Lanning et al., 2020).

Desert ecosystems represent zones that are extremely vulnerable to changes in temperature and anthropogenic disturbances. In desert ecosystems, water is the dominant driver of plant growth, and its absence could affect the survival of desert plants. In water-scarce habitats, water uptake strategies are key characteristics that determine plant survival (Zunzunegui et al., 2022), and awareness of desert plant water-use strategies is indispensable. Although desert plants have developed an array of morphological and physiological characteristics to cope with intensive drought events (Huo et al., 2022), it is crucial for desert plants under conditions of water scarcity to alternate water acquisition sources, modulate water potential to maintain hydraulic conductivity (Huo et al., 2021), and improve WUE. As confirmed in previous studies, desert plants, such as Mongolian pine and *Haloxylon ammodendron*, can alter water sources during the growing season, which is critical for satisfying their water requirements (Grossiord et al., 2017; Wu et al., 2019; Zhou et al., 2019). The two dominant *Haloxylon* species in the Gurbantonggut Desert exhibited distinct water use characteristics and both absorbed water from various sources during the drought and rainy seasons (Dai et al., 2015). Wu et al. (2019) found considerable seasonal variations in the proportion of groundwater contributed to the xylem water of *Haloxylon ammodendron* and *Haloxylon persicum*. Artificial sand-fixing plants in the Tengger Desert absorbed and utilized water from different soil layers during different months (Zhao et al., 2019). Specific monthly variations were detected in the contributions of fog and dew water to diverse plants in the Namib Desert (Wang L. et al., 2019). Plants maximize their exploitation of limited water by altering water sources based on their root characteristics (Wang J. et al., 2019). WUE, defined as the ratio of carbon fixed through photosynthesis to water vapor loss through stomata, is vital for studying the interplay between atmosphere-leaf carbon and water cycle processes and plant survival adaptive responses (Blum, 2009; Shen et al., 2017). A high WUE ensures normal plant physiological life and development (Farquhar et al., 1989; Wang J. et al., 2019; Cao et al., 2020). In recent decades, researchers have identified diverse plant water use strategies that improve WUE by regulating stomatal conductance (Flexas et al., 2016). Water potential is a direct measure of plant water conditions or the degree of water deficit and reflects the plants’ access to water resources and their capacity to address environmental stresses (Zunzunegui et al., 2022). Leaf water potential (*Ψ*), in particular, is the principal driver for stomatal conductance and photosynthetic carbon absorption (Novick et al., 2022), and can effectively be used to evaluate the trade-off between water-source use and plant water deficit (Zunzunegui et al., 2022). In addition, the hydraulic capacity of plants, that is, the water transfer efficiency from the root surface to the leaves, accurately determines *Ψ* (Kangur et al., 2020). In water-stressed environments, isohydric species gradually close their stomata to strictly control *Ψ*, whereas anisohydric species maintain their stomata open, making *Ψ* drop sharply as water availability decreases. It is increasingly acknowledged that the water regulation mechanism is intensely regulated by plant-environment interactions, despite the degree of isohydricity or anisohydricity being an intrinsic characteristic of plants (Ding et al., 2021). The regression slopes of the predawn leaf water potential (*Ψ*_pd_) and midday leaf water potential (*Ψ*_md_) have gained attention for classifying plant water regulation strategies (Martínez-Vilalta et al., 2014; Lanning et al., 2020). Integrating plant water use patterns, WUE, and *Ψ*, to identify plant water use strategies aids in a better comprehension of the diversity of plant species reactions to long-term aridity.

The desert lake-basin region is characterized by a dry weather, sparse rainfall, strong evaporation, and a harsh ecological environment. However, considerable natural vegetation continues to grow from the core of the lake basin to the periphery of the wind-formed dune land, which is distributed in a regular ring belt. Vegetation plays a major role in preserving the ecosystem stability of regions. Regional groundwater is relatively abundant, recharging lake and soil water, and the depth of the water table gradually decreases from the edge of the basin to its interior (Song, 2012). Space-time fluctuations in groundwater level control the regional distribution pattern of plant populations in the area and influence the stability and evolutionary trends of existing vegetation (Song, 2012). A total of 110 perennially waterlogged lakes remain in the Badain Jaran Desert (Wang et al., 2016), creating a unique landscape where lakes and mega-dunes coexist. The periphery of the lake basin predominantly contains *Nitraria* nebkhas (Wang et al., 2016), which attenuate wind and sand flows, trap sand particles, and prevent the forward movement of fluvial sand and land sanding. Furthermore, the groundwater table in the Badain Jaran Desert exhibits significant interannual fluctuations (Huang, 2018), that affect *Nitraria tangutorum* water use; however, the underlying mechanism remains unclear. Therefore, investigating the water use strategies of *N. tangutorum* in the lake basin area of the Badain Jaran Desert is necessary, not only to better appreciate the response of desert plants to variations in water availability but also to assess the sustainability and long-term stability of desert scrubs against the background of future climate changes. Water utilization by plants in the lake basin regions, as the central link of the soil-plant-atmosphere continuum (SPAC), can broaden our knowledge of the key ecohydrological processes in the lake basin regions of desert ecosystems.

This study investigated water use patterns of *N. tangutorum* in the lake-basin area of the Badain Jaran Desert using the stable isotope technique (IsoSource model), and simultaneously explored the spatial and temporal variation of leaf carbon isotope compositions (δ^13^C) and *Ψ* values to synthesize the water use strategies. The objectives of this study were to (1) investigate seasonal variations in water use patterns of *N. tangutorum*, (2) examine the response of WUE to water use patterns, (3) explore the response of *Ψ* to water use patterns, and (4) integrate information on the water use strategies of *N. tangutorum*.

## 2 Materials and methods

### 2.1 Site description

The Badain Jaran Desert (39°04′15″–42°12′23″ N, 99°23′18″–104°34′02″ E) is positioned in the western part of the Inner Mongolia Autonomous Region, China, occupying an area of 5.2 × 10^4^km^2^(Zhu et al., 2010). The Badain Jaran Desert is situated in the center of the Alashan desert, with a typical continental climate, average annual rainfall of 76.9 mm (Ma et al., 2014), and mean annual temperature of 7–8°C. Northwest and west winds prevail perennially, with an annual mean wind speed of 3.0–4.5 m·s^-1^, gradually intensifying from east to west. The desert has a wide range of mega-dunes, generally 150–300 m in height and up to 430 m in height. Numerous small inland lakes are present in the lowlands between the mega-dunes, The lakeside zone consists mainly of saline lakes, and the surrounding zone is dominated by xerophytic and saline-tolerant plants, such as *Phragmites australis*, *Glycyrrhiza uralensis*, *Achnatherum splendens, and N. tangutorum*. Among these, *N. tangutorum* is the most widespread shrub in the lakeshore zone, and is an important building block species in desert and saline regions with high ecological, medicinal, and nutritional value (Liu et al., 2021).

This experiment was performed in the Badan Lake (39°43′19.31″ N, 102°37′1.74″ E, 1218 m) area in the southeastern part of the Badain Jaran Desert. Badan Lake includes the Badan East Lake and Badan West Lake. The former is a freshwater lake with less surrounding natural vegetation owing to tourism development in the area. The latter is a highly saline lake surrounded by shrubs and wetland grasses (Luo et al., 2017). The shrubs are primarily *Nitraria* nebkha species. The groundwater table in the lake basin region is 1.5–2 m, with significant daily and seasonal fluctuations (Huang, 2018). *Nitraria* nebkhas at distances of 20, 50, and 100 m from the Badan West Lake shore were selected as the experimental sample sites.

### 2.2 Sample collection

**Figure 1:**
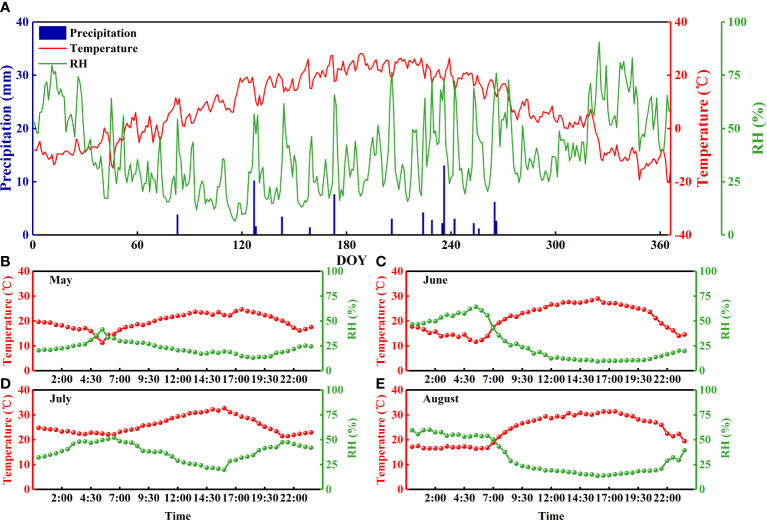
Daily precipitation, air temperature, and relative humidity in the Badain Jaran Desert during 2020 **(A)** and daily variations in air temperature and relative humidity on a typical sunny day during the sampling period **(B-E)**.

Samples were collected from May to August 2020 during the growth period, once in the middle of each month (5.15-5.20; 6.11-6.16; 7.15-7.20; 8.13-8.18) for a total of four collections (**Figure 1**). Suberized and non-green twigs of *N. tangutorum* were clipped into 3–4 cm branch sections, and the phloem tissues were gently removed using tweezers. Then, these twigs were immediately put into airtight glass vials with screw-tops, sealed with Parafilm to avoid evaporation, and stored in a portable freezer at -20°C. Leaves were collected, stored in envelopes, ventilated, and brought back to the laboratory for oven-drying. In parallel with plant sampling, soil samples were acquired around the marked plant individuals by excavating soil profiles, removing the surface dry sand layer (approximately 30–100 cm thick) and collecting at 30 cm intervals from the appearance of a moist sand layer until groundwater emerged (approximately 1.2–1.5 m in depth). If groundwater was not unearthed, eight layers were collected (0–30, 30–60, 60–90, 90–120, 120–150, 150–180, 180–210, and 210–240 cm), and three replicates were collected for each soil layer at each site. Each soil sample was separated into two pieces: one was immediately put into screw-top glass vials, wrapped with Parafilm, and kept at −20°C; the other was put into an aluminum box to measure the gravimetric soil water content (SWC), which was obtained by weighing *in situ* and then brought back to the laboratory to be dried in an oven at 105°C to constant weight. After the samples were collected, the holes were backfilled with sand. Groundwater and lake water were sampled from the wells of pastoralists residing near (approximately 1 km) the experimental site and Badan Lake, respectively, using capped vials sealed with parafilm to prevent evaporation. A total of 819 soil isotope, 108 plant stem, 24 lake water, 12 groundwater, and 108 plant leaf samples were collected.

### 2.3 Isotopic analysis

A cryogenic vacuum distillation system was used to extract water from plant xylem and soil samples. The extraction glass tubes were covered by a heating unit at 95-100°C, and the collection glass tubes were submerged in liquid nitrogen to capture the extracted water vapor. The vacuum pressure was 0.02 Pa. The procedure required 2–3 h depending on the moisture content of the sample. The proportion of water derived from the xylem and soil reached 98%. The hydrogen and oxygen isotope compositions (δD and δ^18^O) of all water samples were determined using an isotope ratio infrared spectroscopy (IRIS) system (LWIA, 912-0008-1001, Los Gatos Research Inc., Mountain View, CA, USA) with measured accuracies of 0.3 ‰ for δD, and 0.1 ‰ for δ^18^O (Zhou et al., 2019). Given that organic contaminants in water derived from plant twigs interfere with the δD and δ^18^O determinations by the IRIS system, the identification and quantification of organic contaminants were performed using spectral contamination post-processing software, and isotopic values of samples were corrected (Schultz et al., 2011; Zhou et al., 2019). The δ^13^C in leaf samples was measured using an isotope ratio mass spectrometer (IRMS) (DELTA V Advantage, Thermo Fisher Scientific, Bremen, Germany) with an accuracy of 0.15 ‰ for δ^13^C (Wang J. et al., 2019). The sample isotopic compositions are expressed as follows:


(1) δX(‰)=(RsampleRstandard−1)×1000

where X represents D, ^18^O or ^13^C, and *R_sample_* and *R_standard_* are the isotopic compositions (D/^1^H, ^18^O/^16^O, and ^13^C/^12^C ratios) of the sample and standard, respectively. The Vienna Standard Mean Ocean Water (V-SMOW) was used as the standard for D and ^18^O and the Vienna Pee Dee Belemnite (V-PDB) was used as the standard for ^13^C.

### 2.4 Quantification of the contribution of potential water sources to plants

The known δD and δ^18^O values of water sources and plant xylem water allow the analysis of water sources absorbed by plants and to quantify the contributions of varying water sources. Based on the similarity in SWC, isotopic composition, and vertical and temporal variations of individual soil layers, the soil layers were aggregated into three main categories: shallow soil layer (0–90 cm), middle soil layer (90–180 cm), and deep soil layer (180–240 cm) to facilitate follow-up analysis and comparison. The IsoSource model (Phillips and Gregg, 2003) was used to assess the proportions of various water sources absorbed by *N. tangutorum*. Considering the hydrogen isotopic fractionation of xerophytic plants (Ellsworth and Williams, 2007; Zhao et al., 2016) and hydrogen isotope biases produced by the cryogenic vacuum distillation system (Chen et al., 2020), we select only δ^18^O values for the analysis and calculation of water sources.

### 2.5 Plant water potential

The *Ψ*_pd_, *Ψ*_md_, and evening leaf water potential (*Ψ*_en_) were measured monthly with a pressure chamber water potential meter (Plant Moisture Stress; Corvallis, Oregon, USA) to assess plant water status. Three healthy, uniformly grown individuals from each site were selected and labeled. From each individual, three well-developed leaves without mechanical damage were cut and immediately placed into the pressure chamber to obtain *Ψ*. *Ψ*_pd_, *Ψ*_md_, and *Ψ*_en_were measured before sunrise, at solar noon, and during the evening, respectively. Each sample site was measured 27 times per day for 3 consecutive days on sunny days in the middle of each month for a total of 4 months, yielding a total of 972 *Ψ* data and 324 each of *Ψ*_pd_, *Ψ*_md_, and *Ψ*_en_ data.

### 2.6 Data analysis

Variations in the SWC and hydrogen and oxygen isotopic composition of soil water (δD_s_, and δ^18^O_s_) with the soil depth and month were analyzed using two-way analysis of variance (ANOVA). Variances in the hydrogen and oxygen isotopic composition of plant xylem water (δD_x_, δ^18^O_x_), δ^13^C, *Ψ* values, and water use were also detected using two-way ANOVA with the month and distance from the lake as the fixed effects. One-way ANOVA was conducted to identify monthly variations in the hydrogen and oxygen isotopic composition of groundwater and lake water (δD_g_, δ^18^O_g_, δD_l_, and δ^18^O_l_). Variations in the δ^13^C, *Ψ*_pd_, *Ψ*_md_, and *Ψ*_en_values among months and distances from the lake were examined using one-way ANOVA with the *post hoc*Tukey’s honestly significant difference (HSD) test. Pearson correlation analysis was performed to identify possible correlations between SWC, δ^13^C, *Ψ*_pd_, *Ψ*_md_, and *Ψ*_en_values and the distance from the lake. Data analyses were conducted using the SPSS software (version 21.0; SPSS Inc., Chicago, IL, USA) and figures were constructed using the Origin 2017 software (OriginLab Corp., Northampton, MA, USA).

## 3 Results

### 3.1 Temporal and vertical variations in SWC

**Figure 2:**
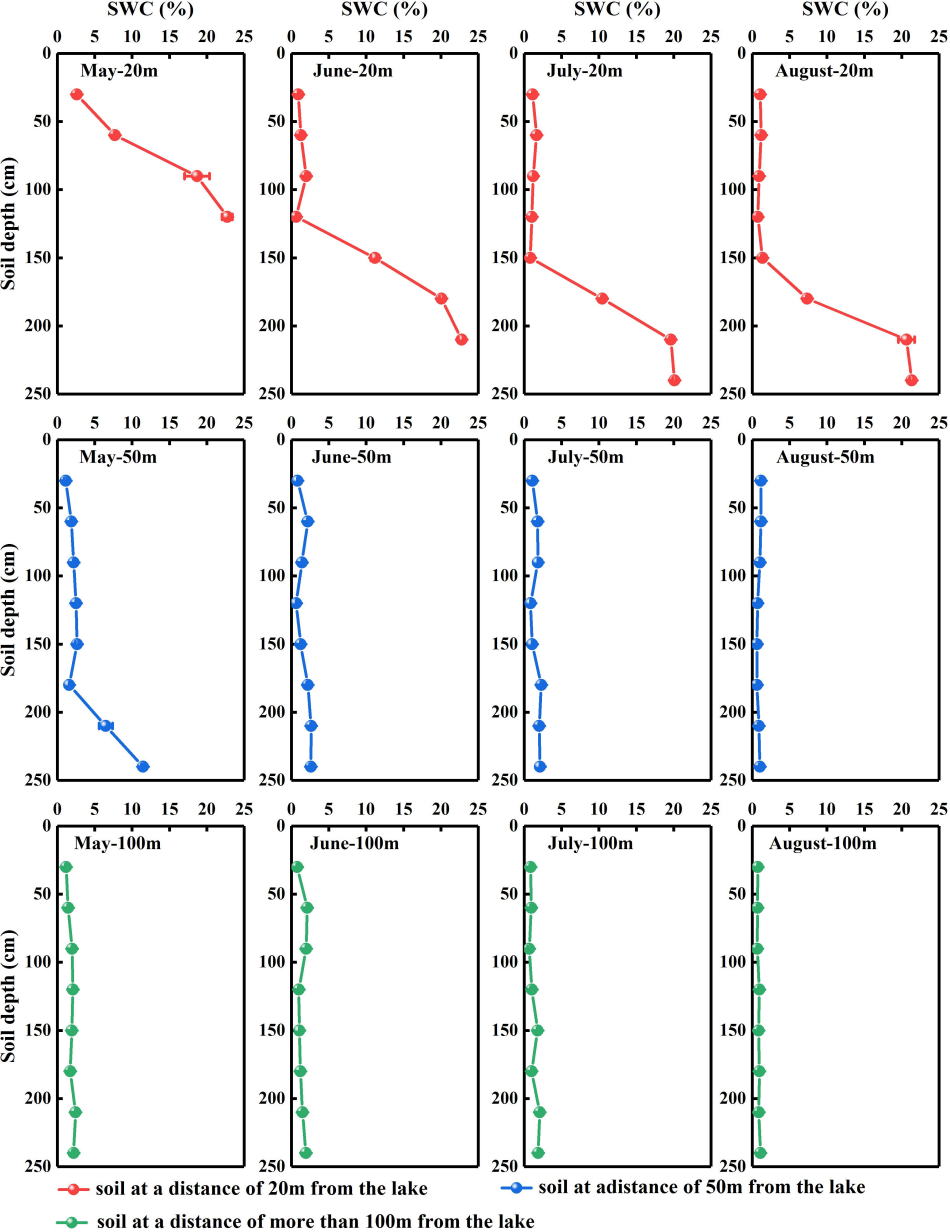
Vertical distribution of the soil water content (SWC) in *Nitraria tangutorum* habitats along different distances from the lake in the Badain Jaran Desert during the growing season of 2020. Rows one to three present the SWC at distances of 20, 50, and 100 m from the lake, respectively. Columns one to four present the SWC in May, June, July, and August, respectively. Data are expressed as means ± 1SE.

SWC in the *N. tangutorum*habitats varied significantly with distance from the lake (**Figure 2**), and a significant negative correlation was identified between SWC and distance from the lake (*r*= −0.427, *P*< 0.001, data not shown). Concurrently, SWC in the *N. tangutorum* habitats exhibited significant variation depending on soil depth (*P*< 0.001) and month (*P*< 0.001) at each study site (**Table 1**). With increasing soil depth, SWC increased gradually with minor undulations (**Figure 2**), and thereafter rapidly increased as the depth increased closer to the groundwater table, especially at 20 m and 50 m from the lake (**Figures 2A–E**). SWC in the shallow, middle, and deep soil layers varied considerably across months (*P*< 0.001) and distances from the lake (*P*< 0.001) (**Table 2**). The average SWC at each studied site was higher in May (20 m: 12.94%; 50 m: 3.64%; 100 m: 1.87%) and June (20 m: 8.40%; 50 m: 1.71%; 100 m: 1.45%) and lower in July (20 m: 7.00%; 50 m: 1.52%; 100 m: 1.27%) and August (20 m: 6.83%; 50 m: 0.90%; 100 m: 0.85%). The overall SWC value in each month was significantly higher at 20 m from the lake than at 50 m and over 100 m from the lake (*P*< 0.001).

**Table 1:** Two-way ANOVA results of the effects of the month, depth, and their interactions on the SWC, δD_s_, and δ^18^O_s_values in the *N. tangutorum* habitats at different distances from the lake.

Position	Source of variation	SWC (%)		δD_s_(‰)		δ^18^O_s_(‰)	
		*F*	*P*	*F*	*P*	*F*	*P*
20 m from the lake	Month	784.933	<0.001	278.261	<0.001	548.394	<0.001
	Depth	1135.907	<0.001	253.003	<0.001	363.017	<0.001
	Month*Depth	95.686	<0.001	31.587	<0.001	28.289	<0.001
50 m from the lake	Month	173.355	<0.001	33.044	<0.001	405.724	<0.001
	Depth	80.017	<0.001	132.778	<0.001	983.955	<0.001
	Month*Depth	45.068	<0.001	26.363	<0.001	132.195	<0.001
100 m from the lake	Month	155.933	<0.001	11.841	<0.001	217.419	<0.001
	Depth	35.927	<0.001	99.073	<0.001	1261.273	<0.001
	Month*Depth	16.191	<0.001	6.983	<0.001	58.306	<0.001

Significance levels: *P*< 0.05.

**Table 2:** Two-way ANOVA results of the effects of the month, distance from the lake, and their interactions on the SWC, δDs, and δ^18^Os of shallow, middle, and deep soils, and δD_x_, δ^18^O_x_, δ^13^C, *Ψ_pd_*, *Ψ_md_*, and *Ψ_en_* of *N. tangutorum*.

		SWC (%)			δD_s_(‰)			δ^18^O_s_(‰)		
Source of variation		shallow	middle	deep	shallow	middle	deep	shallow	middle	deep
Month	*F*	13.687	22.581	48.661	2.334	85.264	84.087	10.395	122.909	98.399
	*P*	<0.001	<0.001	<0.001	0.102	<0.001	<0.001	<0.001	<0.001	<0.001
Distance from the lake	*F*	11.522	73.981	1726.030	13.716	18.259	17.561	3.483	83.922	310.296
	*P*	<0.001	<0.001	<0.001	<0.001	<0.001	<0.001	0.019	<0.001	<0.001
Month*Distance from the lake	*F*	10.798	13.203	2.920	14.250	6.617	5.298	1.204	21.051	15.161
	*P*	<0.001	<0.001	0.068	<0.001	<0.001	0.001	0.311	<0.001	<0.001
		δD_x_(‰)	δ^18^O_x_(‰)	δ^13^C (‰)	*Ψ_pd_* (MPa)	*Ψ_md_* (MPa)	*Ψ_en_* (MPa)			
		
Month	*F*	25.402	6.492	131.192	59.519	46.554	229.205			
	*P*	<0.001	0.006	<0.001	<0.001	<0.001	<0.001			
Distance from the lake	*F*	91.909	48.347	950.926	109.964	18.910	38.112			
	*P*	<0.001	<0.001	<0.001	<0.001	<0.001	<0.001			
Month*Distance from the lake	*F*	3.816	0.912	6.623	1.180	0.673	1.156			
	*P*	0.008	0.503	<0.001	0.350	0.672	0.362			

Significance levels: *P*< 0.05.

### 3.2 Isotopic compositions of xylem water and potential water sources

**Figure 3:**
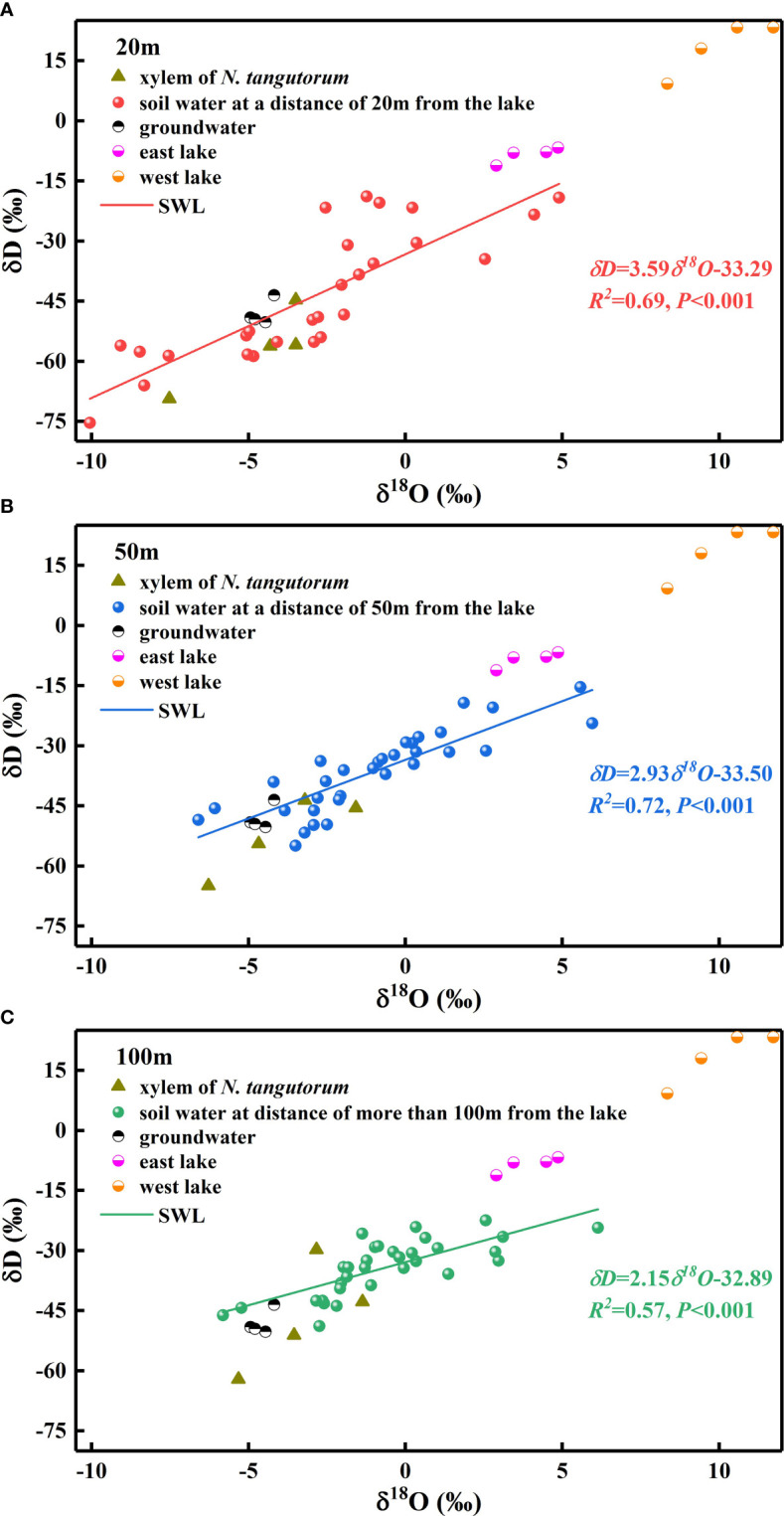
Values of δD as a function of δ^18^O from xylem water in *Nitraria tangutorum* and potential water sources, including soil water, groundwater, and lake water. SWL represents the soil water evaporation line which is fitted based on the isotopic values of soil water **(A) **soil water at a distance of 20 m from the lake: *δD*=3.59*δ^18^O*-33.29, *R^2^ =*0.69, *P<*0.001; **(B) **soil water at a distance of 50 m from the lake: *δD*=2.93*δ^18^O*-33.50, *R^2^ =*0.72, *P<*0.001; **(C)** soil water at a distance of 100 m from the lake: *δD*=2.15*δ^18^O*-32.89, *R^2^ =*0.57, *P<*0.001).

The δD_s_values in soil water showed considerable differences with distance from the lake (*P*< 0.001), which were most enriched at distances greater than 100 m from the lake and most depleted at a distance of 20 m from the lake. In addition, the δD_s_values in soil water differed considerably with month and depth at each experimental site (*P*< 0.001) (**Table 1**). As indicated, the δD_s_values in the shallow soil layers exhibited strongly pronounced monthly variations (*P*< 0.001), with smaller values occurring in May and June and larger values occurring in July and August. Conversely, no statistically significant monthly differences were detected in the δD_s_values of the deep soil layers (*P*> 0.05). Moreover, the δD_s_values declined in fluctuation from the surface of the wet sand layer downward, rather than decreasing monotonically, although a remarkable decrease in the δD_s_values existed from the shallow to middle to deep soil layers (*P*< 0.001). The variation trend in the δ^18^O_s_values of soil water tended to be uniform with the δD_s_values. The slope of the soil water evaporation line (SWL) progressively declined with increasing distance from the lake (**Figure 3**).

**Figure 4:**
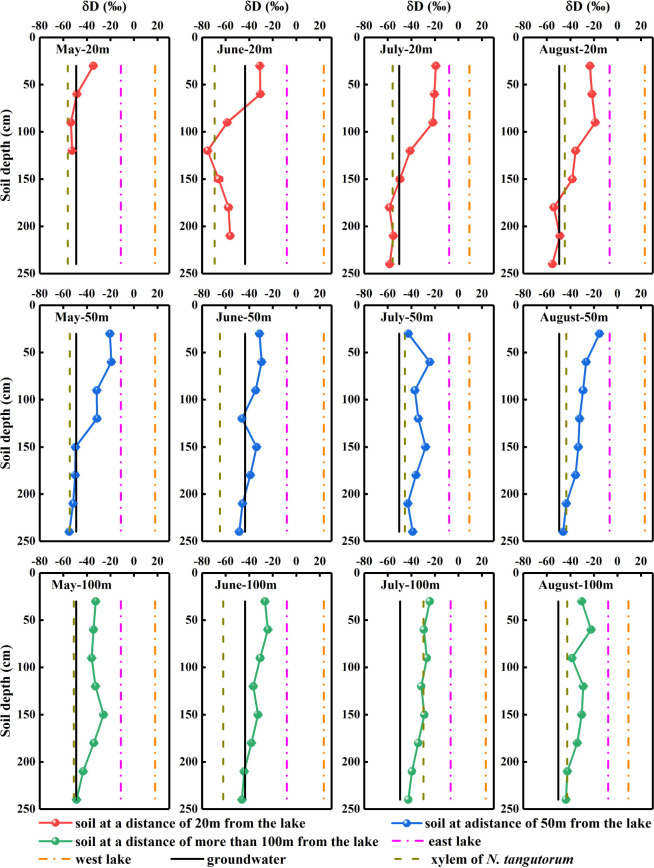
Hydrogen isotopic composition of xylem water and potential water sources for *Nitraria tangutorum*, including soil water, groundwater, and lake water in the Badain Jaran Desert during the growing season of 2020. Rows one to three present the δD values of xylem water and potential water sources at distances of 20, 50, and 100 m from the lake, respectively. Columns one to four present the δD values of xylem water and potential water sources in May, June, July, and August, respectively.

**Figure 5:**
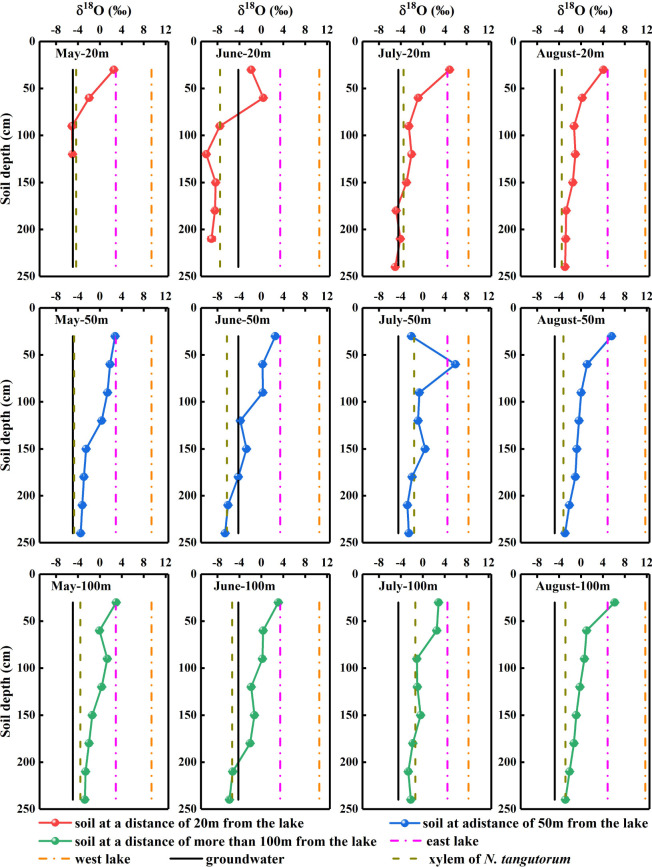
Oxygen isotopic composition of xylem water and potential water sources for *Nitraria tangutorum*, including soil water, groundwater, and lake water in the Badain Jaran Desert during the growing season of 2020. Rows one to three present the δ^18^O values of xylem water and potential water sources at distances of 20, 50, and 100 m from the lake, respectively. Columns one to four present the δ^18^O values of xylem water and potential water sources in May, June, July, and August, respectively.

The δD_g_ and δ^18^O_g_ values of the groundwater varied significantly with time (*P*< 0.05). However, considering the prominent monthly differences in isotopic signatures in other potential water sources, the δD_g_ and δ^18^O_g_ in groundwater were relatively stable (**Figures 4**, **5**).

Significant monthly variations were detected in the δD_l_and δ^18^O_l_values in west lake water (*P*< 0.001). Nevertheless, the east lake water δ^18^O_l_values exhibited notable monthly variations (*P*< 0.001), although no substantial temporal variation was identified in the δD_l_values (*P*> 0.05) (**Figures 4**, **5**).

The δD_x_ and δ^18^O_x_values of *N. tangutorum* both exhibited pronounced differences for two effects: month (*P*< 0.001) and distance from the lake (*P*< 0.01) (**Table 2**). The patterns of monthly variation in the δD_x_ and δ^18^O_x_values were similar, with greater depleted values recorded in May and June and greater enriched values noted in July and August (**Figures 4**, **5**). In addition, the δD_x_and δ^18^O_x_values were the closest to those in the soil water and groundwater (**Figures 4**, **5**), and their scatter points were located near the SWL and groundwater regions (**Figure 3**). Accordingly, *N. tangutorum* may use soil and groundwater as its principal water sources at different distances from the lake.

### 3.3 IsoSource estimation of feasible contributions of potential water sources

**Figure 6:**
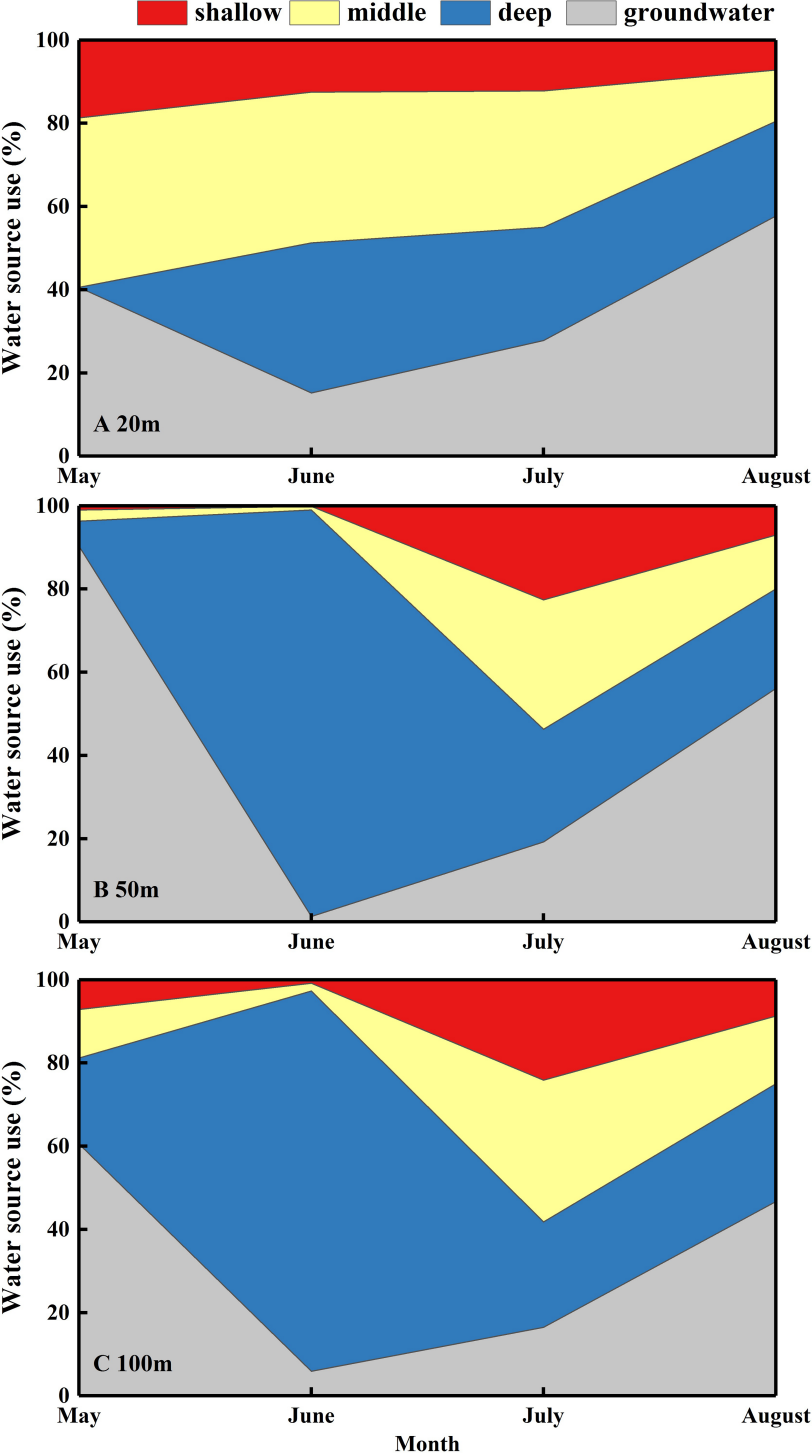
Seasonal variations in the mean percentage of different water sources used by *Nitraria tangutorum* along a distance gradient from the lake in the Badain Jaran Desert during the growing season of 2020. **(A–C)** present data at distances of 20, 50, and 100 m from the lake, respectively. The data were acquired via the Iso-source model: shallow soil layer (0-90 cm), middle soil layer (90-180 cm), deep soil layer (180-240 cm), and groundwater.

Significant differences were identified in the proportion of shallow, middle, and deep soil water, and groundwater that contributed to *N. tangutorum* (*P*< 0.05; **Figure 6**). The IsoSource model revealed that *N. tangutorum* could absorb water from four potential water sources synchronously; however, the relative quantities differed depending on the month and distance from the lake. Groundwater was the primary contributor to *N. tangutorum* xylem water in May (63.8%) and August (53.5%) but not in June and July. In June, *N. tangutorum* depended on deep soil water (75.1%), whereas in July, soil water was mostly collected from different layers (**Figure 6**). From May to June, the percentage of groundwater contributing to *N. tangutorum* xylem water decreased significantly, with a considerable increase in the contribution of deep soil water. The water uptake pattern of *N. tangutorum* at 20 m from the lake differed from those at the other two study sites, especially with respect to the proportion of middle soil water, although *N. tangutorum* at the other two sites exhibited comparable uptake patterns. The proportion of middle soil water contributing to *N. tangutorum* at 20 *m* from the lake was higher than that at the other two study sites (**Figure 6**).

### 3.4 δ^13^C values in plant leaves

**Figure 7:**
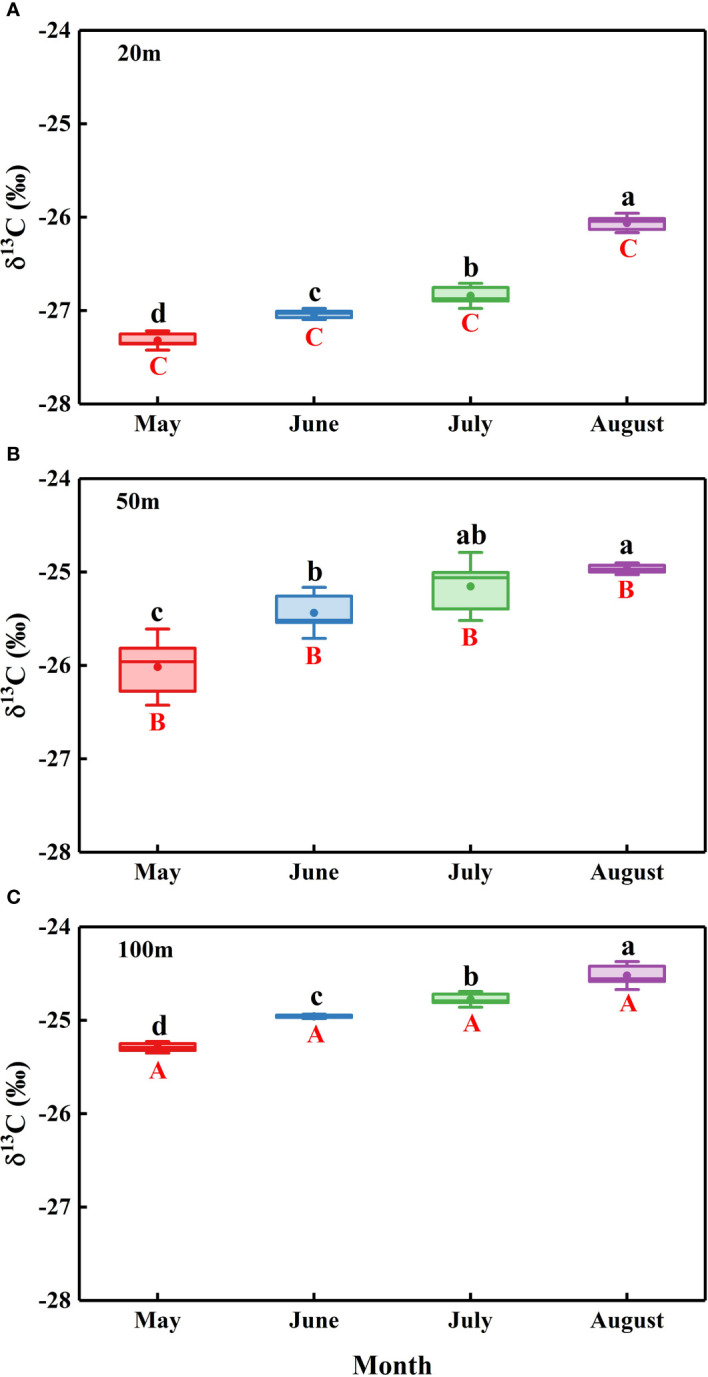
Temporal variations in the leaf δ^13^C of *Nitraria tangutorum* along different distances from the lake in the Badain Jaran Desert during the growing season of 2020. **(A)**, **(B)**, and **(C) **present data at distances of 20, 50, and 100 m from the lake, respectively. Data are expressed as means ± 3SE. The different lowercase letters mean significant differences in leaf δ^13^C among months in each studied site at the level *P*< 0.05. The different uppercase letters express significant differences in leaf δ^13^C across different distances from the lake within a sampling month at the level *P<*0.05.

The *N. tangutorum* leaf δ^13^C values showed remarkable differences between two variables: month (*P*< 0.001) and distance from the lake (*P*< 0.001) (**Table 2**). The mean leaf δ^13^C value of *N. tangutorum* was −25.70 ± 0.15 ‰, ranging from −27.36 ‰ to −24.42 ‰. The leaf δ^13^C values at each experimental site exhibited monthly variations (*P*< 0.001), with highly depleted values in May and June and highly enriched values in July and August. Furthermore, the *N. tangutorum* leaf δ^13^C values showed significant differences among sites at different distances from the lake (*P*< 0.001). The leaf δ^13^C values at 20 m from the lake were generally lower than those of *N. tangutorum* at the other two study sites, and a remarkable positive correlation was identified between leaf δ^13^C values and distance from the lake (*r*= −0.824, *P*< 0.001) (**Figure 7**).

### 3.5 Plant leaf water potential

**Figure 8:**
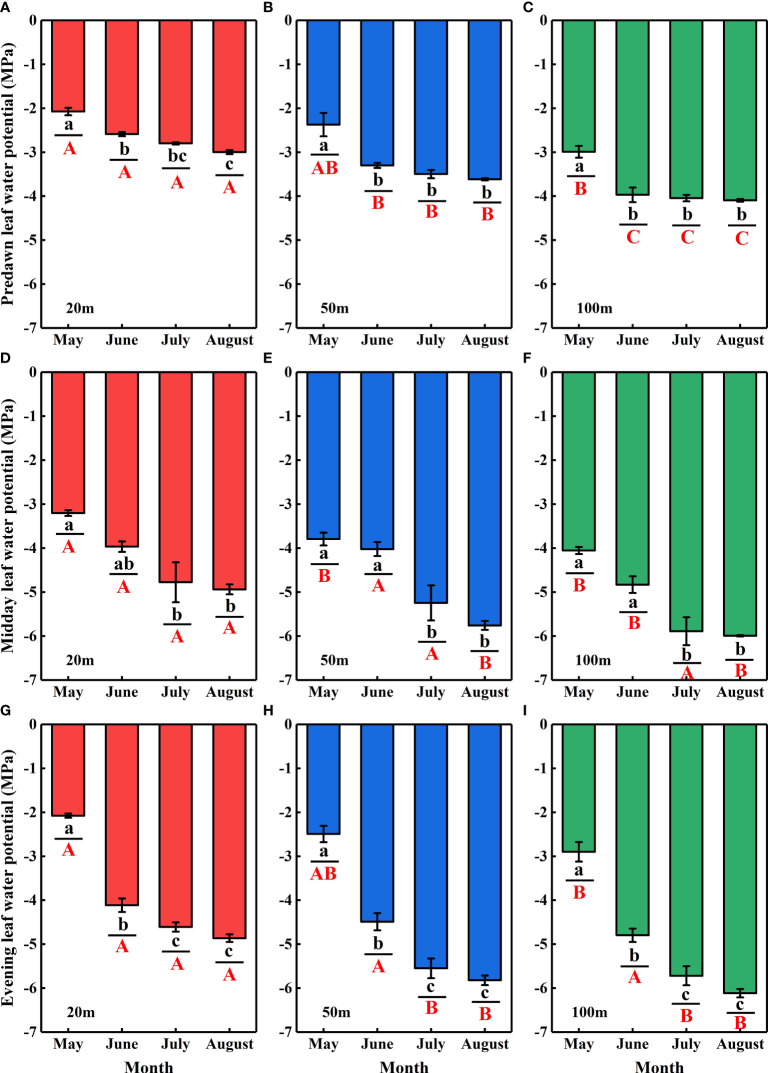
Temporal variations in the predawn (*Ψ*_pd_) **(A-C)**, midday (*Ψ*_md_) **(D-F)**, and evening (*Ψ*_en_) **(G–I)** leaf water potentials for *N. tangutorum*along different distances from the lake in the Badain Jaran Desert during the growing season of 2020. Data are expressed as means ± 1SE. The different lowercase letters express significant differences in plant leaf water potential (*Ψ*) among months in each studied site at the level *P*< 0.05. The different uppercase letters indicate significant differences in plant leaf water potential across different distances from the lake within a sampling month at the level *P*< 0.05.

The *Ψ*of *N. tangutorum* exhibited significant differences with the month (*P*< 0.001) and distance from the lake (*P*< 0.001) (**Table 2**). Significant monthly variations in the *Ψ*_pd_, *Ψ*_md_, and *Ψ*_en_values were present for each experimental site (*P*< 0.01), and notable differences in the *Ψ*_pd_, *Ψ*_md_, and *Ψ*_en_values across studied sites were also identified in most months (*P*< 0.05) apart from the *Ψ*_md_in July and the *Ψ*_en_in June (*P*> 0.05). The *Ψ*_pd_, *Ψ*_md_, and *Ψ*_en_values showed monotonically decreasing temporal patterns, being relatively high in May and June and low in July and August. Moreover, negative correlations existed between the *Ψ*_pd_, *Ψ*_md_, and *Ψ*_en_values of *N. tangutorum*and distance from the lake (*r*= −0.708, *P*< 0.001; *r*= −0.425, *P*< 0.05; *r*= −0.289, *P*= 0.087, respectively) (**Figure 8**).

## 4 Discussion

### 4.1 Relationships among soil water, rainwater, lake water, groundwater, and plant xylem water

The δD values of rainfall in the experimental region varied from −227 ‰ to −20 ‰, whereas the δ^18^O values ranged from −28.5 ‰ to −1.3 ‰, and the equation of the local meteoric water line (LMWL) was *δD*=7.90*δ^18^O*+5.02 (Cao et al., 2021). The δD and δ^18^O values of the soil water, lake water, and groundwater were all situated at the bottom right of this LMWL (**Figure 3**), demonstrating that all these water sources were impacted by evaporation enrichment (Chen et al., 2021). As scarce local rainfall inputs cannot offset for the water loss (Ma et al., 2014; Dong et al., 2016), another recharge source is essential. There are conflicting opinions regarding the regional water cycle (Chen et al., 2014; Zhan et al., 2018; Wang et al., 2021). Some researchers have concluded that local rainfall does not contribute substantially to the regional water systems (Chen et al., 2005; Dong et al., 2016; Jin et al., 2018). Wang et al. (2021) demonstrated that infiltration of local precipitation significantly recharges groundwater. Considering the precipitation isotopic values from previous studies (Jin et al., 2018; Cao et al., 2021) and our results, we inferred that direct infiltration of regional rainfall to recharge the water system exists, but it is not a major recharge source. Lake water is primarily replenished by groundwater in desert hinterlands (Dong et al., 2013; Jiao et al., 2015; Luo et al., 2017). However, the groundwater recharge source is inconclusive; in addition to the modern local precipitation recharge hypothesis mentioned above, various other hypotheses can offer plausible explanations, including the neighboring area recharge hypothesis (Wu et al., 2017; Jin et al., 2018), paleowater recharge hypothesis (Zhao et al., 2012), and remote source recharge hypothesis (Chen et al., 2005; Zhan et al., 2018). Furthermore, year-round variations in lake and groundwater levels in this region are primarily constrained by changes in evapotranspiration, with the highest water levels occurring in winter and spring, followed by a decline, a minimum from July to August, and a continuous increase after September (Lu, 2013). Lake basin vegetation exploits the groundwater, leading to diurnal fluctuations in the water table (Zhang et al., 2020), with the highest water level at approximately 08:00 and the lowest level at approximately 16:00 (Huang, 2018).

The vertical profile of the soil water isotope composition integrates the processes involved in rainfall replenishment, groundwater recharge, blending with pre-existing water, and evaporation (Tang and Feng, 2001; Brooks et al., 2015; Wang J. et al., 2019). Isotopic enrichment of the surface soil (**Figures 4**, **5**) was influenced by evaporation (Zhou et al., 2019), as well as evaporative water vapor condensation (Chen et al., 2014), of which strong evaporation resulted in a lower SWC in this layer (**Figure 2**). In comparison, the deeper soil water contains less heavy isotopes than the shallower layers, primarily attributable to the fact that deep soil layers were less influenced by evaporation and more affected by capillary upward recharge of groundwater containing light isotopes (Wu et al., 2018; Zhou et al., 2019). Additionally, plant water uptake from deep soil layers and fluctuating variations in the groundwater table accounted for the fluctuating changes in SWC in this layer. The SWC and isotopic composition of the deep soils exhibited higher temporal stability than those of the shallow soils, which is in accordance with previous studies (Penna et al., 2013; Yang et al., 2014; Li et al., 2019). The δD_x_and δ^18^O_x_values in *N. tangutorum*xylem water were more similar to those in the soil water and groundwater (**Figures 4**, **5**), exhibiting significant monthly variations according to different uptake ratios. A non-negligible point is that δD_x_values were more depleted than δ^18^O_x_values compared with those of potential water sources, which numerous scholars have suggested may be due to the isotopic fractionation that occurs during root water uptake (Ellsworth and Williams, 2007; Zhao et al., 2016). However, a recent study found that this phenomenon is related to the cryogenic vacuum distillation system used to extract plant xylem water (Chen et al., 2020). This is something that should be explicitly considered when conducting research on plant water sources.

### 4.2 Variations in water use patterns

**Figure 9:**
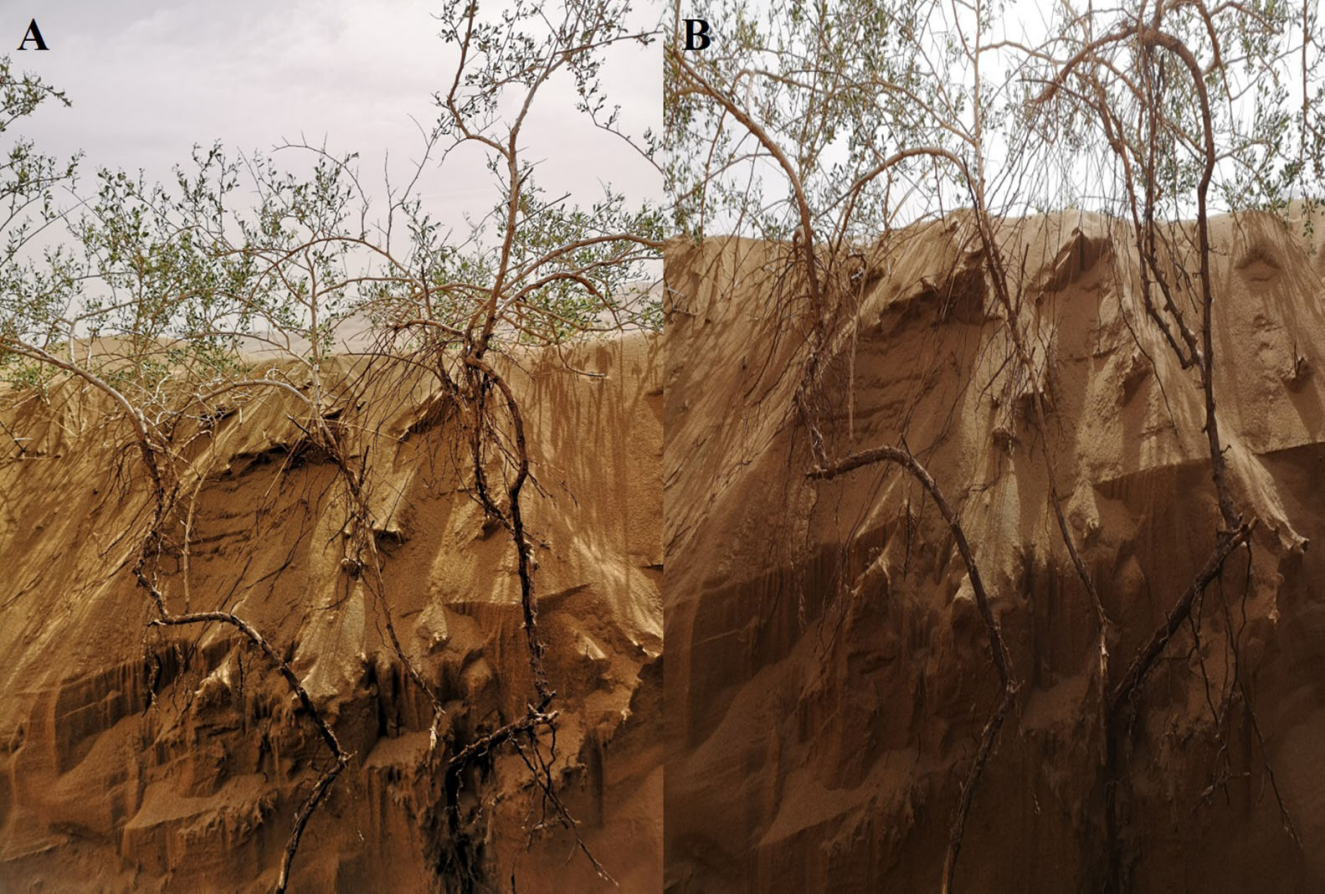
**(A, B)** Photographs of *N. tangutorum*root systems.

In this desert lake basin habitat, fluctuating variations in groundwater levels are responsible for significant differences in *N. tangutorum* water-use patterns and directly affected its growth (Li et al., 2013; Zolfaghar et al., 2015; Zhou et al., 2019). The IsoSource model revealed that *N. tangutorum* could access different water sources simultaneously; however, the percentages of the four potential water sources absorbed by *N. tangutorum* showed remarkable differences over time (**Figure 6**). Overall, *N. tangutorum* mainly assimilated deep soil water and groundwater, which is in accordance with the inference of Dong et al. (2019)regarding the water source of *N. tangutorum*. Due to the shallow groundwater level in May, *N. tangutorum* absorbed groundwater extensively at different distances from the lake during this time (**Figures 6B, C**). The surface soil layer of the *N. tangutorum* nebkha, 20 m from the lake, was the closest to the groundwater table, and groundwater was unearthed in the 120 cm soil layer in May. Soil layers close to the groundwater table remained moist owing to the capillary rise of groundwater (Naumburg et al., 2005); therefore, a sufficient water supply was observed in this layer in May, but the source of its uptake remained groundwater-dependent (**Figure 6A**). As the groundwater table decreased, *N. tangutorum* alternated to drawing on deeper soil water in June to alleviate the water deficit triggered by a falling water table. Upon the depletion of deep soil water (**Figure 2**), this layer no longer met the growth requirements of *N. tangutorum*. Following this, *N. tangutorum* increased the uptake percentage of the middle soil water in July (**Figure 6**), which might be attributed to *N. tangutorum* activating fine roots in this soil layer. Subsequently, as *N. tangutorum* absorbed water from the soil profile, soil water availability was significantly diminished, and water uptake by *N. tangutorum* was hindered. This phenomenon might be primarily due to incomplete root-soil contact and increased hydraulic resistance, which constrainswater movement between the roots and soil (Wu et al., 2019). Temperature and drought stress further result in the dormancy of the surface root system or dehydration and death of fine roots, stimulating root growth in deep soils to absorb deep water sources (Dai et al., 2015). Thus, *N. tangutorum* may have increased the availability of deep soil water and groundwater in August (**Figure 6**) by developing its deep tap roots (Granda et al., 2022). Continuous staged root growth is essential for improving soil WUE and overcoming water uptake restrictions caused by decreased soil water availability (Fernandez and Caldwell, 1975). The maximum growth rate of shrub roots in the arid zones is 3–15 mm·d^-1^(Fernandez and Caldwell, 1975). Variations in water availability may result in variations in root hydraulic architecture to integrate water signals from heterogeneous soil environments and continuously adjust water acquisition strategies (Maurel and Nacry, 2020). The hydraulic lifting effect of *N. tangutorum*’s deep root system should not be ignored when adjusting water-use patterns (Zhao, 2007). Furthermore, in ecosystems where deep-rooted plants coexist with shallow-rooted plants that rely on the hydraulic lifting capacity of deep-rooted plants, shallow-rooted plants can indirectly use deep-water sources. In conclusion, *N. tangutorum* may respond to changes in groundwater depth by adjusting its root distribution to adapt to seasonal shifts in available water sources through rapid growth, activation, or dormancy of the root system at diverse depths (**Figure 9**) to maximize water acquisition (Brooks et al., 2015; Brunner et al., 2015; Granda et al., 2022). This phenomenon of exploiting different water sources in different seasons is prevalent in desert plants ((Dai et al., 2015; Hao and Li, 2021; Granda et al., 2022), which enhances water stress tolerance under natural conditions and serves as an invaluable strategy for allowing desert plants to survive in arid habitats.

*N. tangutorum* in the Ebinur Lake Basin mainly relied on surface soil water in spring, deep soil water in summer, and an increased percentage of middle soil water in autumn (Hao and Li, 2021), which is partially dissimilar to the results of this study. This difference may be primarily relevant to the replenishment of shallow soil water by spring snowmelt in the Ebinur Lake Basin, whereas the extremely low winter precipitation in the Badain Jaran Desert did not replenish shallow soil moisture. *N. tangutorum* on the Loess Plateau predominantly exploits water from the 0–40 cm soil layer, principally because natural precipitation is the only water replenishment source in this habitat (Wang et al., 2013). Consequently, plants adopt various water-use patterns in different habitats to adapt to different water conditions. *N. tangutorum* mainly depended on groundwater for survival in this study (**Figure 6**), and its growth required a suitable groundwater level. Numerous scholars have explored suitable water table depths for *N. tangutorum* between 1.6-4.7 m using different methods (Ma and Wang, 2005; Song, 2012; Zhong et al., 2002). The groundwater in the lake basin region of the Badain Jaran Desert is buried at a depth of approximately 1.5–2 m (Huang, 2018). However, *N. tangutorum* scrub dunes in the lake-basin region were taller, generally at 3.0–5.0 m or higher, essentially exhibiting higher height with increasing distance from the lake. This finding, coupled with groundwater level fluctuations, may have contributed to the monthly variations in the water utilization patterns of *N. tangutorum*.

### 4.3 Variations in water use efficiency

WUE is intimately associated with water-use patterns, revealing the competitive strategies of plants under restricted water availability (Su and Shangguan, 2020). To a certain extent, δ^13^C values can reflect the degree of plant water stress and WUE (Easlon et al., 2014; Mohale et al., 2014). Numerous studies have reported a significant positive correlation between δ^13^C values and WUE (Ehleringer and Cooper, 1988; Ebdon et al., 1998). In this study, the space-time variations in the WUE (δ^13^C) of *N. tangutorum* were fundamentally demonstrated as a possible response to the groundwater table, showing a highly significant positive correlation with the groundwater burial depth (Dong et al., 2019). As the groundwater table decreases, decreasing soil water availability or increasing temperatures throughout the growing season can cause structural and physiological changes in plants (Tong et al., 2019), which can alter their water-use strategies and improve WUE. Examples of these changes include smaller and thicker leaves, smaller specific leaf area, and higher leaf nitrogen content, thus achieving higher photosynthesis rates while reducing the evaporative area of a single leaf (Wang et al., 2017); development of longer fine roots, increased specific root length, and decreased water consumption (Li et al., 2023); and control of stomatal openness (Graham and Zhang, 2014), among others. As drought progresses, some plants display greater stomatal conductance variation, resulting in an increased plant WUE (Lázaro-Nogal et al., 2013; Jiang et al., 2021). Some species obtain high carbon assimilation rates and maintain high WUE by reducing water loss through reduced stomatal conductance (Yang et al., 2018). Moreover, C_3_ plant tissues subjected to water stress have higher WUE or δ^13^C values than non-water stressed plant tissues (Santesteban et al., 2015; Bchir et al., 2016). In addition to water conditions, light and temperature can affect plant WUE (Zhu et al., 2015). The monthly variations in the WUE (δ^13^C) of *N. tangutorum* in this research confirmed that the differences in light and water due to seasonal changes affected the plant WUE (Shen et al., 2017).

**Figure 10:**
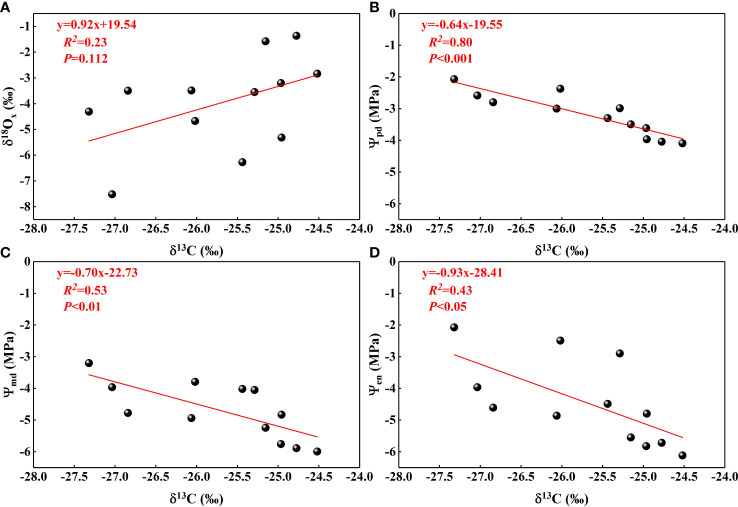
Relationships between leaf δ^13^C and δ^18^O values from xylem water **(A)**, *Ψ*_pd_ **(B)**, *Ψ*_md_ **(C)**, and *Ψ*_en_ **(D)**.

Plant leaf δ^13^C values provided information about photosynthesis and transpiration (Shen et al., 2017), the main processes of water consumption in plants, while δ^18^O_x_values in plants xylem water in this study indicated the sources of water. There was a non-significant positive correlation between the two (**Figure 10A**), indicating that *N. tangutorum* exhibited some increase in photosynthesis and WUE when using shallow water sources. The fractionation patterns of δ^18^O values in plant water were driven by the transpiration process, which required us to obtain the δ^18^O values of different plant parts and fit them with leaf δ^13^C values to determine the dominant water use process. Although the δ^18^O fractionation patterns of water in different parts of the plant were not obtained in this study, they ware supplemented with *Ψ* at different times that were closely related to transpiration. The negative correlation between plant leaf δ^13^C values and *Ψ* (**Figures 10B–D**) suggested that efficient plant water utilization is closely related to stomatal conductance and is dominated by transpiration processes. The highly significant negative correlation between *Ψ_pd_* and δ^13^C values also suggested that water utilization is more efficient when plants are water-deprived. Although plant transpiration water consumption accounts for the majority of plant water consumption, photosynthesis water consumption should not be neglected. In the future, we should clarify the transpiration and photosynthesis water consumption and confirm the existence of stem water storage to establish the plant water balance equation in this habitat by determining the water source, water absorption, and the water consumption of each process, among others, and to more accurately determine the plant WUE and the dominant process of water use.

### 4.4 Variations in plant water potentials

The *Ψ*of *N. tangutorum* decreased according to month (**Figure 7**) in response to an intensifying water deficit due to the declining groundwater table, which is related to *N. tangutorum* water use patterns. As water stress intensifies and the effective soil water is depleted, plants experience increasing difficulty in obtaining water. Under such circumstances, some plants exhibit sensitive stomatal behavior to keep a certain *Ψ* by rapidly diminishing stomata opening, which reduces the photosynthetic rate, and it is typically referred to as isohydric behavior. In contrast, other plants leave a certain extent of stomata open to preserve a high photosynthetic rate, which is known as anisohydric behavior (Lanning et al., 2020). Martínez-Vilalta et al. (2014)developed a theoretical model by measuring *Ψ*_pd_ and *Ψ*_md_ to classify plants into four categories: strictly isohydric, partially isohydric, strictly anisohydric, and extremely anisohydric. However, in reality, plant water regulation always occurs on a continuum from conservative isohydric to adventurous anisohydric regulation. In the present study, based on the *Ψ*_pd_ and *Ψ*_md_values, *N. tangutorum* mainly used anisohydric regulation and gradually shifted to isohydric regulation as soil water availability decreased. This phenomenon explains the space-time changes in the WUE (δ^13^C) of *N. tangutorum* in this study. Long (2022)also discovered that the water regulation strategy of *N. tangutorum* gradually switched from anisohydric to isohydric regulation with decreasing SWC, and that its lethal mechanism changed from hydrodynamic failure into carbon starvation.

The *Ψ*_en_of *N. tangutorum* increased in May compared to that *Ψ*_md_, whereas it remained low in June–August. This finding was predominantly associated with the daily temperature variation in different months in this region, with lower evening temperatures in May and higher evening temperatures in June–August. In addition to water potential, hydraulic conductivity is a crucial indicator for analyzing water regulation strategies. To better understand the water regulation strategies of *N. tangutorum* under water stress conditions, future analyses should be conducted in conjunction with hydraulic characteristics such as hydraulic conductivity. Root, stem, and leaf hydraulic conductivity all affect the *Ψ*. If the stem exhibits high water flow resistance, the root hydraulic conductivity characteristics have a restrictive impact on *Ψ* (Turner, 1982). Even in cases of high stem water potential, the *Ψ* will probably remain low when the inner hydraulic conductivity of the leaf remains low (Nissanka et al., 1997).

## 5 Conclusions

In this study, the spatial and temporal variations in the water use patterns, WUE, and *Ψ* of *N. tangutorum* were integrated to investigate water use strategies of the species in the lake basin regions of the Badain Jaran Desert. Soil water availability in the study region is constrained by periodic fluctuations in the groundwater level associated with precipitation recharge, pre-existing water mixing, and evapotranspiration processes. In the desert lake basin habitats, *N. tangutorum* mainly depends on groundwater for survival and may adapt to variations in water availability by assigning root functions at different depths, and absorbing different water sources in different months. With decreasing water availability, *N. tangutorum* continuously increased WUE and reduced *Ψ* to ensure a constant water supply and reduce the impact of a water deficit. As water availability continued to decrease, the water regulation mechanism of *N. tangutorum* shifted from adventurous anisohydric regulation to conservative isohydric regulation to maintain its survival. This study highlights the diversity of desert plants responses to changes in water availability and presents valuable information for further investigation of the groundwater-lake-mega-dune-vegetation hydrological cycle in the Badain Jaran Desert.

## Data availability statement

The raw data supporting the conclusions of this article will be made available by the authors, without undue reservation.

## Author contributions

JQ and JS proposed the research and designed the experiments. JQ conducted field and laboratory measurements, analyzed the data, and wrote the manuscript. The other co-authors participated equally in the investigation, data analysis, and manuscript preparation and editing. JS, BJ, and CZ secured the funding. All authors contributed to the article and approved the submitted version.
